# A Physiology-Based Pharmacokinetic Framework to Support Drug Development and Dose Precision During Therapeutic Hypothermia in Neonates

**DOI:** 10.3389/fphar.2020.00587

**Published:** 2020-05-13

**Authors:** Anne Smits, Pieter Annaert, Steven Van Cruchten, Karel Allegaert

**Affiliations:** ^1^Neonatal Intensive Care Unit, University Hospitals Leuven, Leuven, Belgium; ^2^Department of Development and Regeneration, KU Leuven, Leuven, Belgium; ^3^Drug Delivery and Disposition, Department of Pharmaceutical and Pharmacological Sciences, KU Leuven, Leuven, Belgium; ^4^Applied Veterinary Morphology, Department of Veterinary Sciences, University of Antwerp, Wilrijk, Belgium; ^5^Department of Pharmaceutical and Pharmacological Sciences, KU Leuven, Leuven, Belgium; ^6^Department of Clinical Pharmacy, Erasmus MC-Sophia Children's Hospital, Rotterdam, Netherlands

**Keywords:** physiology-based pharmacokinetic modelling, neonate, pharmacokinetics, drug metabolism, therapeutic hypothermia

## Abstract

Therapeutic hypothermia (TH) is standard treatment for neonates (≥36 weeks) with perinatal asphyxia (PA) and hypoxic–ischemic encephalopathy. TH reduces mortality and neurodevelopmental disability due to reduced metabolic rate and decreased neuronal apoptosis. Since both hypothermia and PA influence physiology, they are expected to alter pharmacokinetics (PK). Tools for personalized dosing in this setting are lacking. A neonatal hypothermia physiology-based PK (PBPK) framework would enable precision dosing in the clinic. In this literature review, the stepwise approach, benefits and challenges to develop such a PBPK framework are covered. It hereby contributes to explore the impact of non-maturational PK covariates. First, the current evidence as well as knowledge gaps on the impact of PA and TH on drug absorption, distribution, metabolism and excretion in neonates is summarized. While reduced renal drug elimination is well-documented in neonates with PA undergoing hypothermia, knowledge of the impact on drug metabolism is limited. Second, a multidisciplinary approach to develop a neonatal hypothermia PBPK framework is presented. Insights on the effect of hypothermia on hepatic drug elimination can partly be generated from *in vitro* (human/animal) profiling of hepatic drug metabolizing enzymes and transporters. Also, endogenous biomarkers may be evaluated as surrogate for metabolic activity. To distinguish the impact of PA *versus* hypothermia on drug metabolism, *in vivo* neonatal animal data are needed. The conventional pig is a well-established model for PA and the neonatal Göttingen minipig should be further explored for PA under hypothermia conditions, as it is the most commonly used pig strain in nonclinical drug development. Finally, a strategy is proposed for establishing and fine-tuning compound-specific PBPK models for this application. Besides improvement of clinical exposure predictions of drugs used during hypothermia, the developed PBPK models can be applied in drug development. Add-on pharmacotherapies to further improve outcome in neonates undergoing hypothermia are under investigation, all in need for dosing guidance. Furthermore, the hypothermia PBPK framework can be used to develop temperature-driven PBPK models for other populations or indications. The applicability of the proposed workflow and the challenges in the development of the PBPK framework are illustrated for midazolam as model drug.

## Introduction

### Perinatal Asphyxia

Despite advances in perinatal medicine, the incidence of perinatal asphyxia (PA, a clinical condition comprising perinatal hypoxia, hypercarbia and combined metabolic and respiratory acidosis) has not decreased in the last decade (Albrecht et al., 2019). PA occurs in 5.4/1,000 live born neonates, with 1.8/1,000 live births diagnosed with hypoxic–ischemic encephalopathy (HIE) (Pokorna et al., 2015). Hypoxic–ischemic injury to the brain may result in persistent neurological impairment. This brain injury is secondary to the hypoxic–ischemic event as well as the reoxygenation–reperfusion after resuscitation (oxidative stress with production of free radicals) (Solevag et al., 2019). Besides the immediate cell death (necrosis), delayed death (apoptosis) of neuronal cells can occur after a hypoxic insult. This apoptosis process substantially contributes to the permanent brain damage after PA (Solevag et al., 2019). Cerebral palsy, epilepsy, mental retardation, cerebral visual impairment, learning, and behavioral problems can occur as long term consequences (Albrecht et al., 2019).

**Figure 1:**
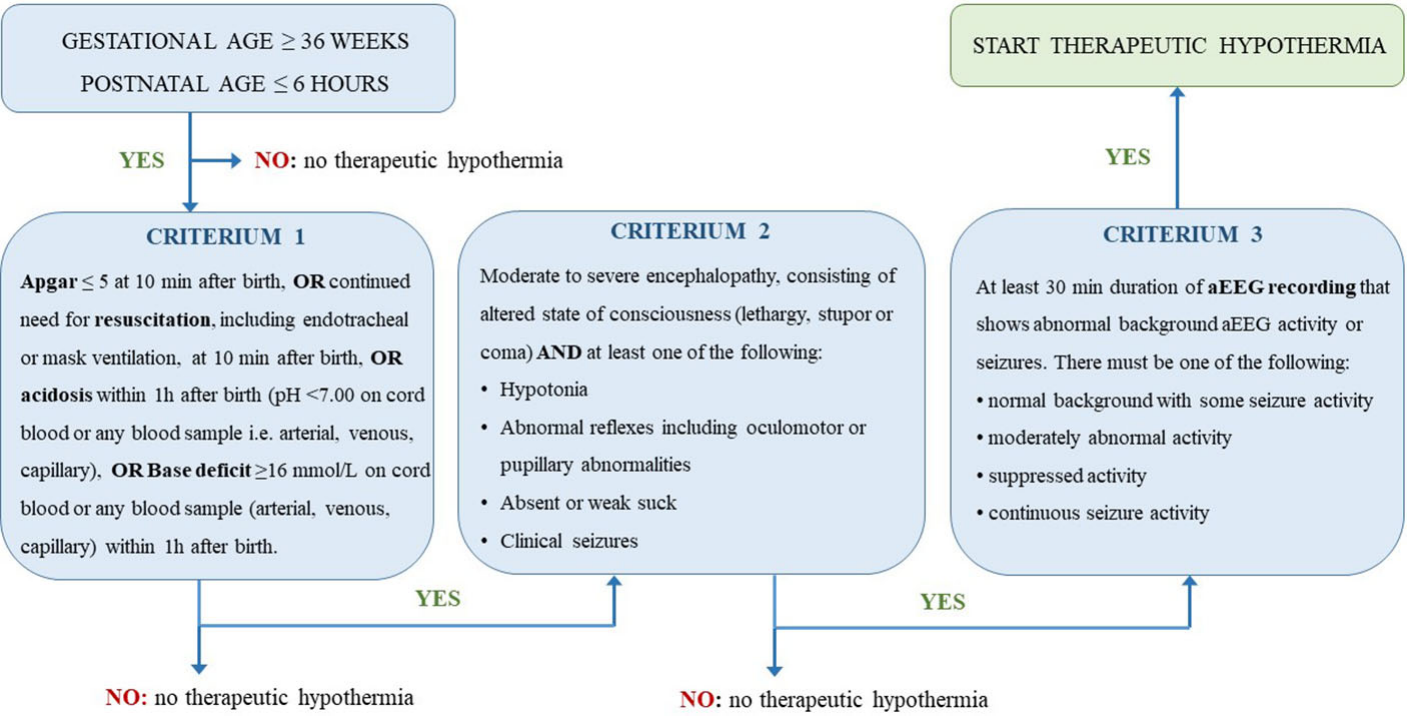
Visual presentation of the sequential evaluation of the criteria used in the TOBY study to determine if therapeutic hypothermia needs to be started in neonates (Azzopardi et al., 2008).

Intensive care unit admission with multiple drug treatment is often needed in neonates presenting PA. Whole body cooling therapy, also known as therapeutic hypothermia (TH), is used as standard of care in neonates with PA and HIE since 2010 (NICE, 2010). Criteria to start this treatment slightly differ between studies. In **Figure 1**, criteria as used in the TOBY trial are visually presented (Azzopardi et al., 2008). Therapeutic hypothermia (*i.e.* core body temperature of 33.5°C for 72 h) reduces mortality and neurodevelopmental disability (number needed to treat equal to 7) in term and late preterm neonates with moderate-to-severe HIE, when initiated before 6 h of age. These 6 h constitute the therapeutic window to reduce delayed brain damage (Azzopardi et al., 2009; Jacobs et al., 2013; Azzopardi et al., 2014; Albrecht et al., 2019). The benefits of hypothermia on survival and neurodevelopment outweigh the short-term adverse effects. These benefits are due to reduced metabolic rate and decreased neuronal apoptosis. Since both TH and PA influence physiology, they are also expected to alter pharmacokinetic (PK, concentration–time) and pharmacodynamic (PD, concentration–effect) processes.

### Neonatal Pharmacology: Driven by Maturational and Nonmaturational Factors

For each of the four main PK processes, *i.e.* drug *A*bsorption, *D*istribution, *M*etabolism, and *E*xcretion (ADME), remarkable qualitative and quantitative differences have been described between neonates and adults or between neonates and other pediatric age groups (Van Den Anker et al., 2018). After oral ingestion, a drug will be absorbed from the gastro-intestinal tract (mainly small intestine) and appears in the blood compartment. The absorption profile will differ in case other routes of administration are used (*e.g.* intramuscular, transdermal) (Kearns et al., 2003). When the intravenous (iv) route is used for drug administration, the drug appears entirely and directly in the blood. From the blood compartment, drugs will be distributed to other organs and tissues. Most drugs are metabolized to inactive metabolites, which are excreted from the body. While metabolic clearance is mainly hepatic, elimination clearance is predominantly renal. The neonatal population is characterized by prominent changes in growth and maturation, impacting these ADME processes (developmental pharmacology) (Smits et al., 2013).

The knowledge on *maturational covariates* that impact neonatal drug disposition has substantially increased in the last decades. Drug absorption after oral ingestion depends on maturational changes in *e.g.* gastric pH, gastric emptying, intestinal transit time and absorption rate. Based on clinical literature data of selected compounds after iv and oral dosing, Somani et al. performed a population PK analysis to evaluate changes in oral drug absorption postnatally. They concluded that the maturational changes in oral drug absorption occur within the first week after birth and are drug-independent (Somani et al., 2016). Besides drug administration by oral route, also nonenteral routes might be used. Absorption after intramuscular (im) administration is difficult to predict in neonates due to reduced skeletal muscle blood flow, and inefficient muscular contractions on the one hand, and the presence of a higher capillary density in skeletal muscles in this patient population on the other hand (Carry et al., 1986; Tayman et al., 2011). For transdermal absorption, developmental changes in skin physiology (*e.g.* thinner stratum corneum in preterms) need to be taken into account (Allegaert et al., 2017a). Sublingual and rectal routes for drug administration have the advantage of bypassing the first-pass effect, but are only rarely used in neonates. For rectal administration, the extensive exposure variability in neonates is a major disadvantage. Age-dependent alterations in body composition and protein binding during early life, affect drug distribution, while systemic drug metabolism changes due to maturation of *e.g.* transporters, liver enzymes as well as plasma protein binding (Allegaert et al., 2017b). For the cytochrome P450 (CYP) enzymes involved in phase 1 metabolism, each isoenzyme displays its own expression and activity ontogeny profile. At birth, CYP3A7 is the most abundant CYP iso-enzyme, but during the first year of live CYP3A7 activity decreases while CYP3A4, the major iso-enzyme for drug metabolism in adults, displays increased activity during early life (De Wildt et al., 1999; Ince et al., 2013). This maturation of CYP, but also glucuronidation capacity in neonates, depends on both postnatal (PNA) and postmenstrual age (PMA) (Allegaert et al., 2009). This has been documented for different compounds, including propofol (Allegaert et al., 2007; Smits et al., 2013). Besides metabolic elimination, also renal elimination (glomerular filtration and tubular functions) undergoes maturation. Ontogeny of glomerular and tubular functions is driven by age (gestational age, GA and PNA), and PNA also determines hemodynamic alterations in cardiac output and regional perfusion. Glomerular filtration rate (GFR) hereby increases rapidly during the first 2 weeks PNA and reaches adult capacity at the age of 8–12 months (Kearns et al., 2003; Rhodin et al., 2009). The above-mentioned developmental processes illustrate that neonatal physiology directs neonatal pharmacology and in part accounts for the large inter- and intraindividual variability in neonatal PK/PD (Smits et al., 2013).

Besides maturational covariates like age, weight or ontogeny of ADME-relevant proteins, variability in drug disposition also depends on *nonmaturational covariates*. Particularly, environmental factors (*e.g.* nutrition), genetic polymorphisms, treatment modalities [*e.g.* extra-corporeal membrane oxygenation (ECMO), TH, drug–drug interactions], and diseases (*e.g.* asphyxia, sepsis, meningitis) need to be considered (Allegaert et al., 2017a; Allegaert et al., 2017b). ECMO is a life-saving therapy for well-selected critically ill neonates. As recently summarized by Raffaeli et al., the ECMO modality itself can, in addition to the severe disease conditions for which ECMO is indicated, increase the volume of distribution (Vd) for lipophilic, and to a lesser extent, also hydrophilic compounds in neonates. Increased Vd as well as clearance (CL) during ECMO therefore requires adequate adaptations of both loading and maintenance doses to keep drug exposure within therapeutic ranges (Raffaeli et al., 2019). Another example of nonmaturational factors for drug disposition is disease condition. As part of the NeoMero trials in neonates with late onset sepsis (NeoMero-1) and/or meningitis (NeoMero-2), Germovsek et al. reported a (model-based) penetration of meropenem, a broad-spectrum carbapenem, into the cerebrospinal fluid (CSF) of 8% in neonates (Germovsek et al., 2018). This overall low value was attributed to the absence of meningeal inflammation in many of the NeoMero-1 patients without meningitis. However, this percentage rose with increasing CSF protein, with even 40% penetration predicted at a protein concentration of 6 g/L, *i.e.* when inflammation of the meninges is present (Germovsek et al., 2018). For asphyxiated neonates treated with TH, both the underlying pathophysiology as well as the hypothermia treatment are considered as nonmaturational factors impacting drug disposition. Besides PK, also PD can be altered during hypothermia. To illustrate this, a prospective study in neonates receiving TH and treated with phenobarbital for seizures, no (clinically relevant) effect of TH on phenobarbital PK was seen, and observed responsiveness was 66%. In addition, a reduced transition rate from continuous normal voltage (CNV) to discontinuous normal voltage background pattern on amplitude integrated electroencephalography was observed, attributed to the add-on neuroprotection of phenobarbital in case of CNV pattern (Van Den Broek et al., 2012).

Unfortunately, optimal dosing recommendations for most conditions in this neonatal subpopulation are often not available. Tools to support prediction of personalized dosing in the setting of PA in need for TH, might bridge this gap. (Roka et al., 2007; Tortorici et al., 2007; Van Den Broek et al., 2010). In this review, an overview will be provided of the current knowledge on the impact of hypothermia on the ADME processes, as well as remaining knowledge gaps. Subsequently, a stepwise approach to develop a framework towards precision dosing in the very specific setting of neonatal PA with TH will be described, as well as challenges and applicability of this proposal.

## Hypothermia and Neonatal Drug Disposition

### Currently Available PK Studies in Neonates Undergoing Therapeutic Hypothermia

In order to provide an overview of the currently available published PK data of individual compounds in neonates undergoing whole body hypothermia (WBH), a structured search was performed on PubMed on 21 August 2019 with ‘pharmacokinetics' and ‘hypothermia' as search terms. When ‘newborn' was added, this resulted in 82 hits. Titles and—when appropriate—abstracts and full papers were assessed with the intention to illustrate the diversity of compounds with PK data in this specific subcategory of neonates (**Table 1**). The bulk of currently available data relates to antibiotics (n = 5, gentamicin, amikacin, ampicillin, amoxicillin, benzyl-penicillin), antiepileptic or potential neuro-protective drugs (n = 8, phenobarbital, topiramate, erythropoietin or darbepoetin, midazolam, melatonin, bumetanide, lidocaine), or morphine, reflecting the currently most commonly used drugs in these patients. The majority of these compounds are administered by iv route (n = 12), while two drugs are administered by enteral route (melatonin, topiramate).

**Table 1:** Overview on the pharmacokinetics (PK) of drugs in neonates undergoing therapeutic hypothermia (TH), obtained by a structured Pubmed search.

PMID	Drug	Log P	Study related aspects and findings
**30300445**	gentamicin	−3.1	N = 12 WBH neonates, 2.2, SD 0.7 ml/min/kg
**29781085**	gentamicin	−3.1	pooled data, 8 studies in WBH and non-WBH neonates, trough (+35%) higher, clearance (−31%) lower in WBH neonates.
**26763684**	gentamicin	−3.1	N = 47 WBH neonates during and after cooling, +29% clearance after cooling.
**25225917**	gentamicin	−3.1	N = 41 WBH neonates, external validation of the PIMD 23553582 dosing regimen.
**25070069**	gentamicin	−3.1	N = 15 WBH *vs* 19 non-WBH asphyxia neonates, elimination half-life 9.57 *vs* 7.01 h, +36%.
**23702622**	gentamicin	−3.1	N = 29 + 23 WBH neonates, 5 mg/kg.q24 h to 36 h dosing strategy, elevated trough (>2 mg/l) 38 to 4%.
**23553582**	gentamicin	−3.1	N = 29 WBH neonates, clearance 0.111 × (weight/3.3 kg)^0.75^ × (1/crea, mg.dl)^0.566^.
**23503448**	gentamicin	−3.1	N = 16 WBH neonates *vs* 7 non-cooled asphyxia neonates, clearance -25 %, elimination half-life +40 %.
**28993332**	amikacin	−7.4	N = 56 WBH neonates, compared to noncooled, nonasphyxia cases, clearance −40.6%.
**28226400**	ampicillin	1.35	N = 13 WBH neonates, clearance lower (−66%), volume of distribution higher (+30%) compared to historical data.
**28555724**	amoxicillin	0.87	N = 125 WBH neonates, during and after cooling, clearance increases after cooling (0.26 to 0.41 l/h, +58%).
**29378710**	benzyl-penicillin	1.83	N = 41 WBH neonates, clearance 5.33 l/h.70 kg (system information from PIMD 28555724).
**29357720**	phenobarbital	1.47	N = 26 WBH neonates, clearance is lower (−15%), because disease severity in WBH neonates affects clearance (−55%).
**23254984**	phenobarbital	1.47	N = 39 asphyxia neonates, 20/39 underwent WBH, hypothermia was not a significant covariate for clearance.
**23018530**	phenobarbital	1.47	N = 31 WBH neonates, typical newborn (3.5 kg) 17.2 ml/h, weight as covariate (kg^0.81^).
**21371018**	phenobarbital	1.47	N = 19 WBH neonates, half-life 173.9, SD 62.5 h
**19744111**	topiramate	−0.7	N = 13 WBH neonates, (oral), half-life 35.58, SD 19.3 h (deep *vs* mild hypothermia, 30–33 *vs* 33–34°C, half-life + 38%)
**31336401**	topiramate	−0.7	N = 52 WBH neonates, (oral), PK during and after cooling (clearance +21% after cooling)
**28099423**	erythropoetin	n.a.	N = 47 WBH neonates, pooled from previous studies, clearance 0.0289 l/h, weight^0.75^ covariate (1.27)
**23008465**	erythropoetin	n.a.	N = 24 WBH neonates, nonlinear pharmacokinetics (half-life 7.2–18.7 h, clearance 15.6–7.7 ml/h.kg)
**25989868**	darbepoetin alfa	n.a.	N = 16 WBH neonates exposed, clearance 0.0465 l/h
**25996892**	darbepoetin alfa	n.a.	N = 30 WBH neonates, different doses (placebo, 2 or 10 µg/kg <12 h and 7 days), linear pharmacokinetics
**24288267**	midazolam	4.33	N = 9 WBH neonates, half-life 13 (2.75–50.52) h; clearance: 2.57 ml/kg/min, renal or hepatic impairment affects clearance
**31256150**	midazolam+phenobarbital	4.33/1.47	68 WBH neonates, phenobarbital coexposure increases midazolam clearance (factor 2.3) during cooling
**30734962**	melatonin	n.a.	N = 5 WBH neonates, (oral), 0.21, SD 0.07 l/h.
**26189501**	bumetanide	2.6	N = 13 WBH neonates, 1 noncooled, mean clearance 0.063 l/h, weight^1.7^ as covariate
**23303304**	lidocaine	2.44	N = 22 WBH neonates, compared to N = 26 historical noncooled asphyxia neonates, clearance −24%
**18381513**	morphine	0.89	N = 10 WBH neonates, and N = 6 non-cooled asphyxia neonates, clearance 0.69 vs 0.89 ml/min.kg (−22%)
**27225747**	morphine	0.89	N = 20 WBH neonates, 0.765 *vs* 1.42–1.53 l/h in non-asphyxia, noncooled historical neonates (−40 and −50% respectively)

Some of these PK estimates were compared with either noncooling asphyxia cases, nonasphyxia term cases or were compared to PK estimates (paired) after the hypothermia episode. The PMID number, the drug involved, its Log P value (PubChem), study related aspects and key findings have been listed. We refer the interested reader to the original publications (by PMID number) for further details. N, number; PK, pharmacokinetics; SD, standard deviation; WBH, whole body hypothermia.

### Knowledge on the Impact of Hypothermia on ADME Processes in Neonates

Neonatal pharmacology and its inter- and intraindividual variability are driven by maturational physiology and is further affected by nonmaturational, pathophysiological changes including asphyxia, and interventions like TH (Allegaert et al., 2017b). The phenotypic PK hereby reflects the impact of the (patho)physiology of the individual newborn (renal, hepatic, and/or circulatory dysfunction), as well as the impact of TH (reduced energy needs) (Zeilmaker et al., 2018). This setting is not unique, since similar to *e.g.* patent ductus arteriosus, where both the (patho)physiology of the clinical syndrome (vascular steal phenomenon, with lung overflow, and systemic hypoperfusion) (Hundscheid et al., 2019) and its pharmacological management (ibuprofen, indomethacin or paracetamol) will affect the phenotype (Allegaert et al., 2013; Cristea et al., 2019).

However, the impact of PA and TH on the PK has not a single, uniform pattern, but will also depend on drug (Log P, molecular weight, protein binding), individual patient (weight, extent of renal or hepatic impairment) and ADME (*e.g.* extraction ratio) characteristics (Pokorna et al., 2015; Zeilmaker et al., 2018). This is also reflected in **Table 1**, in which a lower gentamicin (Choi et al., 2018) or amikacin (Cristea et al., 2017) clearance is related to TH and/or PA (−31%, −40.6%), with an increase in clearance (+29%) over time (after TH, highly correlated with increasing PNA) (Bijleveld et al., 2016), and weight and/or creatinine as covariates (Frymoyer et al., 2013; Cristea et al., 2017). A similar pattern can be observed for different *β*-lactam antibiotics (ampicillin, amoxicillin, benzyl-penicillin) with reduced clearance during controlled hypothermia (−66%) (Cies et al., 2017) and a subsequent increase in clearance (+58%) over time (Bijleveld et al., 2018). In contrast, phenobarbital clearance is either similar (Shellhaas et al., 2013) or only marginally (Pokorna et al., 2019) decreased and more affected (−55%) by disease severity (Pokorna et al., 2019). Topiramate data suggest an impact of temperature (deep *versus* mild hypothermia, half-life +38% in deep hypothermia) and time (clearance +21% after rewarming) (Filippi et al., 2009; Marques et al., 2019). 1-hydroxy-midazolam elimination clearance is decreased (−26%) during hypothermia (Favie et al., 2019), while midazolam clearance in neonates is not influenced by TH but affected by phenobarbital coadministration (Favie et al., 2019) and renal or hepatic impairment (Welzing et al., 2013). At least for the TH aspect, this is similar to findings on the absence of an effect of moderate hypothermia on midazolam clearance in adults after resuscitation (Bastiaans et al., 2013). Lidocaine clearance is decreased (−22%) when compared to a nonasphyxia, noncooled cohort of term neonates (Van Den Broek et al., 2013). Morphine clearance is affected when compared to noncooled asphyxia cases (−22%) (Roka et al., 2008) or nonasphyxia term neonates (−40 to −50%) (Frymoyer et al., 2017). Since the different compounds are eliminated by different routes, the compound specific observations do reflect the specific impact of TH and/or PA on the elimination route, or the impact of these covariates on drug disposition (ADME).

Changes in *absorption* may affect the subsequent patterns of distribution, metabolism, and excretion. This is because barriers (like intestinal mucosa, skin) can block, delay, or limit (first pass metabolism) drugs during passage. Consequently, TH and PA likely alter drug absorption, resulting in more variable drug plasma concentrations after enteral administration during TH. Data on absorption of drugs in a setting of asphyxia and TH in neonates are at present limited to topiramate and melatonin (Filippi et al., 2009; Balduini et al., 2019; Marques et al., 2019). Other pieces of information relate to data on (patho)physiological changes, like intestinal permeability or intestinal blood flow. The lactulose and renal lactulose/L-rhamnose excretion as markers of maturational intestinal permeability provide evidence for a higher permeability up to at least day 9 in term neonates following perinatal asphyxia (Beach et al., 1982). Celiac and mesenteric artery flow remained low during hypothermia with a significant increase after rewarming (peak systolic velocity celiac 0.63 to 0.77, + 22%; mesenteric 0.43 to 0.55 m/s. +28%), in line with a simultaneous increase in left ventricular output (106 to 149 ml/kg/min, +40%) (Sakhuja et al., 2019). Along the same line, bosentan absorption in critically ill neonates with pulmonary hypertension is delayed and steady state concentrations were only achieved from postnatal day 5 onwards (Steinhorn et al., 2016). A relevant physiological factor that influences drug absorption from an intramuscular injection site is the blood flow in the muscle mass. This may also be compromised in newborns with poor peripheral perfusion and low-cardiac-output. Thomson et al. reported on the variability in gentamicin exposure in young infants with suspected severe sepsis due to unpredictability in the rate and extent of absorption after intramuscular administration (Thomson et al., 2003).

*Drug distribution* also depends on patient- and drug-related characteristics, which may change during TH and PA. *In vitro* studies with a large set of structurally diverse compounds (n = 19, Log P values between −3 and +8) on the impact of low temperature on the fraction unbound for human plasma suggest that hypothermia itself—if any—only marginally affects protein binding (Ryu et al., 2018). However, the blood pH itself is affected by hypothermia since carbon dioxide (CO2) partial pressure will be lower, and pH is higher during hypothermia, while the clinical indication for TH usually is associated with initial metabolic acidosis and pCO2 retention (Van Den Broek et al., 2010; Pokorna et al., 2015). Since hypothermia affects blood gas parameters, it is important to take this into account when interpreting clinical as well as research data in this population (Groenendaal et al., 2009). Besides the blood pH, patient-related characteristics like general and regional blood perfusion, plasma protein composition, or body composition including water sequestration are also different in this subpopulation. Furthermore, these characteristics in an individual patient may evolve over time, with *e.g.* albumin concentration dependent on the fluid status or HIE severity (Muniraman et al., 2017) and changes in alfa-1 glycoprotein (GA, type of delivery, time after surgical or inflammatory event) (Calder et al., 2012; Anell-Olofsson et al., 2018). As a result, Vd can decrease, increase, or remain unchanged. The available observations in human neonates are very limited, but include *e.g.* the ampicillin observations (Vd +30%) (Bijleveld et al., 2018).

Third, hypothermia has been reported to alter *drug metabolism*. The knowledge on drug metabolism during hypothermia remains fragmented and mainly relates to nonclinical studies and observed increased drug and metabolite plasma concentrations in human patients when routine dosing is applied. Since the rate of most enzymatic processes is lower at decreased temperature, hypothermia may cause adverse effects due to higher than anticipated dose-normalized exposure. Furthermore, due to reduced hepatic blood flow, metabolic clearance of drugs with a high hepatic extraction ratio (propofol −25%) is more affected by hypothermia compared to low clearance drugs (Van Den Broek et al., 2010; Pokorna et al., 2015). This illustrates that both reduced liver blood flow and decreased metabolic capacity (*i.e.* intrinsic CL) have to be considered during hypothermia. As summarized by Tortorici et al. using nonclinical data, hypothermia can decrease the systemic CL of drugs metabolized by cytochrome P(CYP)450, by 7–22% per °C below 37°C (Tortorici et al., 2007). Along the same line, mild hypothermia (35.4°C instead of 37°C) altered midazolam pharmacokinetics (decrease in metabolite formation clearance, −11.1%/°C) in healthy adult volunteers (Hostler et al., 2010). In contrast, and reflecting the complex interaction of disease and intervention, there was no additional effect of moderate hypothermia on midazolam clearance in adults after resuscitation (Bastiaans et al., 2013). In neonates, the available evidence suggests that 1-hydroxy-midazolam elimination clearance (renal route) is affected (−26%) during hypothermia (Favie et al., 2019), while midazolam clearance in neonates is affected by phenobarbital coadministration (Favie et al., 2019), renal or hepatic impairment (Welzing et al., 2013). Lidocaine clearance is decreased (−22%) when compared to nonasphyxia, not cooled term neonates (Van Den Broek et al., 2013), but these phenotypic observations may also in part depend on plasma binding proteins (pH, alpha-1 glycoprotein concentration). Phenobarbital clearance is mainly affected by disease severity (Pokorna et al., 2019). Morphine clearance, in part depending on phase 2 metabolism (glucuronidation, sulfation) is decreased in neonates during TH when compared to noncooled asphyxia cases (−22%) or noncooled historical controls (−40 to −50%) (Roka et al., 2008; Frymoyer et al., 2017). Interestingly, peak and mean bilirubin over time are lower in infants with HIE when compared to controls, irrespective of the use of TH, although this likely relates to decreased heme-oxygenase activity and not glucuronidation (Dani et al., 2018).

**Figure 2:**
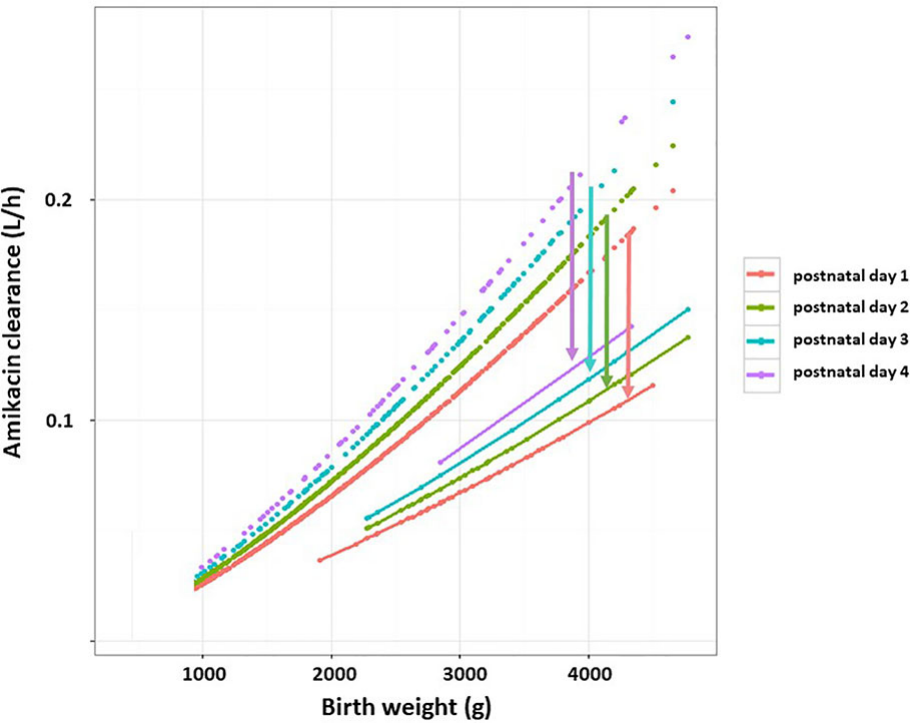
Estimates of amikacin clearance (L/h) trends in early neonatal life based on pooling of reported datasets (dashed lines). There is a maturational trend in clearance related to birth weight (g) and postnatal age (PNA, days 1,2,3,4, as reflected by the colors) compared to a subgroup of term neonates undergoing therapeutic hypothermia as treatment for perinatal asphyxia (solid lines) (Cristea et al., 2017). The arrows indicate the difference in clearance between both cohorts for the respective PNA. Adapted from De Cock et al., antibiotic dosing in pediatric critically ill patients, Chapter in *Antibiotic pharmacokinetic/pharmacodynamic considerations in the critically ill*, Springer Nature Signapore 2018:239–263, with permission from Springer Nature (De Cock et al., 2018).

Finally, hypothermia reduces *renal drug excretion*. For drugs eliminated by glomerular filtration rate (GFR) in neonates with PA and TH, studies consistently showed that elimination is significantly (−30 up to −40%) reduced, with an increase after finalization of the TH (> 72h) (Mark et al., 2013; Ting et al., 2015; Bijleveld et al., 2016; Cristea et al., 2017; Choi et al., 2018). A similar pattern can be observed for the different *β*-lactam antibiotics (ampicillin, amoxicillin, benzyl-penicillin). A retrospective analysis on gentamicin trough levels in 55 neonates included in the CoolCAP trial (RCT design with selective head cooling, not whole-body hypothermia) suggests that the reduced temperature did not affect gentamicin clearance, but the renal function impairment (creatinine) correlated (r2 = 0.36) with the trough level (Liu et al., 2009). To further illustrate the combined impact of maturational changes and PA + TH, we have plotted the amikacin clearance trends in early neonatal life based on pooling of reported datasets (**Figure 2**). There is a maturational trend in clearance, related to birth weight and PNA (days 1,2,3,4, as reflected by the colors) compared to a subgroup of term neonates undergoing TH as treatment for PA (Cristea et al., 2017; Smits et al., 2017). The impact of hypothermia on renal tubular functions is still unexplored.

### Ongoing Drug Development Plans as Add-On Interventions to Whole Body Hypothermia to Improve Outcome

While there is robust evidence that TH reduces mortality and improves the likelihood of normal neurological outcome following moderate to severe asphyxia in (near)term neonates, there is still relevant mortality and morbidity. Consequently, there is a very active research line investigating add-on interventions as adjuvant to further improve this setting (Jacobs et al., 2013). Because of the short timelines (< 6 h PNA) and the fact that the diagnosis is commonly only made at delivery, recruitment for such studies is demanding, while a relevant number of products are at present considered.

In an attempt to provide an overview on the currently ongoing drug development plans and compounds to improve the outcome after moderate and severe perinatal asphyxia, we have explored the clinicaltrial.gov website, the databank of the European Medicine Agency and performed a nonsystematic search on PubMed (asphyxia, newborn) on adjuvant or primary interventions. This resulted in a relevant number of drug development plans and ongoing studies for Argon, Xenon, VH-N439, cannabidiol, allopurinol, 2-iminobiotin and melatonin, besides stem cells related interventions (https://clinicaltrials.gov/; https://www.ema.europa.eu/en). The PubMed search also resulted in these hits, with magnesium sulfate, topiramate, and N-acetylcysteine as examples of additional products or interventions (Mcadams and Juul, 2016; Albrecht et al., 2019; Solevag et al., 2019). Based on the different biochemical processes occurring during the different stages of hypoxic–ischemic injury, Solevag et al. divided these (promising) add-on therapies in targeting the acute injury phase (*to limit necrosis, e.g.* allopurinol, melatonin, xenon, argon, magnesium), the subacute injury phase (*to limit apoptosis*, *e.g.* N-acetylcysteine, 2-iminobiotin, cannabidiol, and doxycycline) and the repair phase after PA (*to improve recovery*, *e.g.* erythropoietin, mesenchymal stem cells, topiramate, and memantine) (Solevag et al., 2019). At present, TH is the only established therapy in the subacute phase of PA related brain injury.

For the drug development programs of the compounds listed above, different animal models were used, while early biomarkers to assess potential efficacy also differ (biochemical biomarkers, MRI imaging or electrophysiological signals). This setting impairs drug development programs and very likely will benefit from a master protocol approach and a multi-stakeholder platform as has been reported for other pediatric orphan settings like pediatric oncology (Woodcock and Lavange, 2017; Khan et al., 2019). In addition, an optimal timing, dosing, and administration route need to be determined for these neuroprotective compounds, considering the cooling and rewarming phases that may impact their disposition. To achieve this, these product development programs will likely also benefit from an effective pharmacometric framework for this specific indication in a special subgroup of neonates. Physiology-based PK (PBPK) modeling is a promising tool for this setting (Smits et al., 2019).

### PBPK Modeling as Tool for Personalized Medicine

As the combination of maturational and nonmaturational covariates results in extensive variability in neonatal drug disposition, safe and effective pharmacotherapy requires proper investigation of drugs in this vulnerable population and driven by their unique (patho)physiology. Nevertheless, most drugs are currently still developed based on adult pathophysiology and driven by adult indications (Allegaert et al., 2018). In addition, knowledge on specific neonatal pathophysiological processes and disease mechanisms is still limited compared to other populations. These shortcomings urgently highlight the need for structured and innovative approaches to make progress in the field of neonatal pharmacology.

A promising tool is PBPK modeling which combines drug-specific information with available physiological data of the population of interest. PBPK aims to generate a predictive model which mathematically describes underlying physiological and biochemical processes explaining ADME of the studied compound. The species to be modeled is represented as a set of various compartments, representing organs or tissues, which are interconnected by the venous and arterial blood (Khalil and Laer, 2011). In general, drug transfer between the blood and the various tissue compartments is captured in equations, taking into account blood flow to the specific tissues, tissue volumes, the change in drug concentration over time, and the tissue–plasma partition coefficients (Michelet et al., 2017). The concept itself has already been introduced years ago but has been applied to the pediatric population only the last 2 decades. For neonates, knowledge on ontogeny and maturation of different systems has to be integrated in this approach (Michelet et al., 2017). In conclusion, since PBPK modeling allows for capturing the rapidly changing, time-dynamic physiological maturation of the neonatal period, combined with the impact of nonmaturational factors, it is considered as a crucial tool to improve neonatal pharmacotherapy. More specifically, PBPK facilitates *drug dose prediction* and *(pediatric) drug development programs*, including clinical trial development and conduct (Khalil and Laer, 2011; Michelet et al., 2017).

#### PBPK Modeling to Support Drug Dose Precision and Drug Development During Hypothermia in Neonates

At present, the predictive performance of neonatal PBPK models is limited, both for drugs metabolized by CYP enzymes as well as for renal excreted drugs (Zhou et al., 2016; Zhou et al., 2018). Knowledge on system ontogeny and on the effect of nonmaturational covariates on drug disposition is needed to further improve neonatal PBPK models (Smits et al., 2019). Therapeutic hypothermia in neonates is one of the conditions for which development of a PBPK model is urgently needed. First, by considering TH as a nonmaturational covariate, a PBPK model would enable more individualized drug exposure prediction. Although TH often is standardized, clinical care and supportive therapy including antibiotics, antiepileptics, inotropics and analgo-sedatives in these neonates may differ between institutions, which can influence outcomes in the setting of TH. PBPK modeling can be useful in optimizing dosing guidelines for these supportive therapies. Second, although TH is effective, further outcome improvement in these patients is feasible with add-on pharmacotherapy (section *Ongoing Drug Development Plans as Add-on Interventions to Whole Body Hypothermia to Improve Outcome*). Accurate dose prediction of these promising and neuroprotective add-on compounds would benefit from a PBPK framework. The strategy to develop such a framework will be discussed in the next section.

### Strategy for PBPK Framework Development

#### General, Multidisciplinary, and Translational Approach

**Figure 3:**
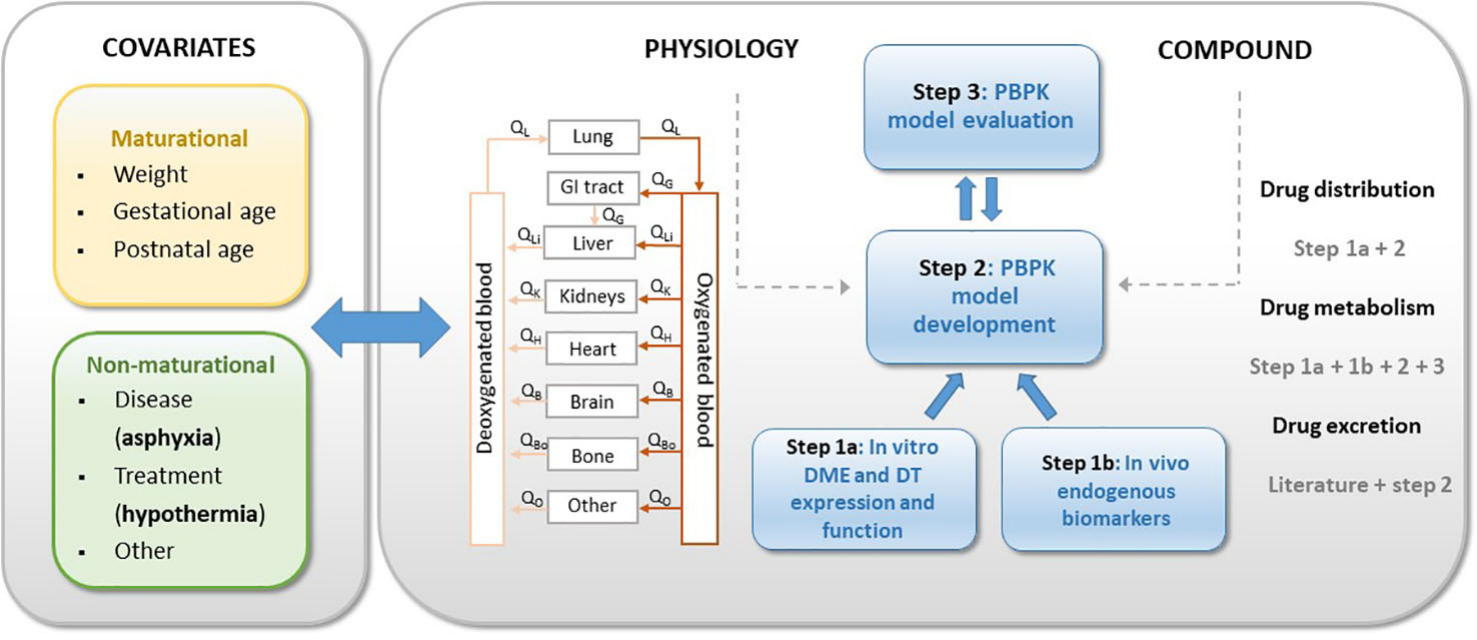
Strategy for neonatal hypothermia PBPK framework development. PBPK, physiology-based pharmacokinetics; DME, drug metabolizing enzymes; DT, drug transporters.

Based on the summary provided in the section *Hypothermia and Neonatal Drug Disposition* of this review, the impact of hypothermia on renal drug elimination in neonates seems relatively well covered, while knowledge regarding the effect of hypothermia on drug metabolism is limited and fragmented. Since pharmacotherapy during hypothermia is mainly intravenously, the absorption phase is not present and only needs to be considered in case of biliary excretion. Therefore, new data generation and research in this field should focus on the impact of hypothermia on drug metabolism. This additional knowledge is essential in the development of a PBPK modeling platform that allows for describing and predicting the impact of TH on drug disposition in asphyxiated neonates. In the current section, we aim to provide a strategy on how to develop this PBPK framework, based on nonclinical and clinical data. We will hereby integrate an approach to generate new data on the main knowledge gap, *i.e.* the impact of hypothermia on drug metabolism. Overall, a multidisciplinary and translational approach is needed, combining *in vitro* as well as *in vivo* animal and human data. **Figure 3** presents the approach, in which physiological information of the population of interest (*i.e.* neonates), including the impact of (non)maturational covariates (*i.e.* asphyxia, hypothermia) and compound-specific information are combined (**Figure 3**). To assess the impact of hypothermia on drug metabolism (**step 1**), controlled hypothermia can be applied on cryopreserved (neonatal) hepatocytes from human donors and animals to determine the impact of temperature on expression and function of drug metabolizing enzymes (DME) and drug transporters (DT) (**step 1a**). Selection of the optimal juvenile animal model is of utmost importance. As will be discussed more in detail later in this review (section *In Vivo Studies in Animal Models*), the newborn conventional piglet is already a well-established translational model for neonatal asphyxia due to the natural occurrence of the condition in this species, but also due to its large body size at birth which allows for surgical application and sampling of the animals (Cheung et al., 2011). As the Göttingen Minipig is the most commonly used pig strain in nonclinical drug development (Bode et al., 2010; Colleton et al., 2016), this model could provide very useful data for pediatric drug use and development. Next, exploration of the utility of endogenous biomarkers reflecting DME and DT, both in the human *in vivo* neonatal setting as well as in the minipig, is useful to gain new data for the PBPK model development (**step 1b**). Endogenous biomarkers are considered as surrogate for determinants of hepatic drug elimination by *e.g.* CYP3A4 (4*β*-hydroxycholesterol, 4*β*-OHC; 6*β*-hydroxycortisol, 6*β*-OHF) and OATP (organic anion transporting polypeptide)1B1/3 (coproporphyrin I and III, CPI-III). Combining the data on drug disposition achieved in steps 1a and 1b, with available literature data on physiology and on *in vivo* PK observations, allows for developing the model (**step 2**). PBPK-based predictions of dose–exposure relationships for selected model compounds (selected based on physicochemical/PK characteristics and clinical relevance) should be the final aim of this step. Primarily renally cleared compounds *versus* primarily hepatic cleared compounds need to be considered as candidates. Subsequently, available *in vivo* PK data in neonates with PA and TH, as well as data collected in an experimental *in vivo* setting, *i.e.* in a neonatal/juvenile animal model (including the selected model compounds), can be used to evaluate the model (**step 3**). Verification of PBPK-based predictions against clinical data, followed by model optimizations can be used as a strategy to achieve a robust PBPK framework. This strategy requires collaboration of (human and veterinary) clinicians, modelers and basic scientists. The nonclinical steps hereby allow for distinguishing the impact of asphyxia *versus* hypothermia, which is not feasible in the clinical setting. In the section *Endogenous Biomarkers as a Surrogate for CYP3A and OATP1B1/3 Activity*, an overview of current knowledge and limitations on selected human endogenous biomarkers, of relevance for further research in TH condition, will be provided followed by current evidence and challenges on *in vitro* (section *In Vitro Approaches to Explore the Impact of TH on PK in Neonates*) and animal *in vivo* (section *In Vivo Studies in Animal Models*) research useful for the development of a robust neonatal PBPK hypothermia framework.

#### Endogenous Biomarkers as a Surrogate for CYP3A and OATP1B1/3 Activity

Population-specific biomarker values and their covariates are useful in the development of PBPK models for compounds undergoing metabolism. The above mentioned biomarkers, plasma 4*β*-OHC (and 4*β*-OHC/cholesterol ratio), urinary 6*β*-OHF (and 6*β*-OHF/cortisol ratio) and, CPI-III will be further discussed. **Table 2** presents biomarker values of healthy (pre)term neonates as retrieved in English papers of which full text was accessible. Values assessed in pathologic conditions, as well as values collected in the same subjects but after the neonatal period, were excluded. To assess these biomarkers in neonates with PA and TH, collaboration with multicenter trials, preferably following a master protocol approach as mentioned in the section *Ongoing Drug Development Plans as Add-On Interventions to Whole Body Hypothermia to Improve Outcome*, is suggested due to the orphan condition of this disease.

**Table 2:** Summary of biomarker values (surrogates for CYP3A and OATP1B1/3 activity) of healthy (pre)term neonates as retrieved in English papers of which full text was accessible.

Biomarker and reference	Number of neonates	PNA (days)	Biomarker value^a^	Sample
**4*β*-OHC**				
Nylen et al., 2011	8	1	12 ng/ml (QR 5,4)	Cord blood
	14	2-3	20.2 ng/ml (QR 4,7)	Venous blood
**4*β*-OHC/cholesterol ratio**				
Nylen et al., 2011	8	1	0.19 (QR 0.07)	Cord blood
**6*β*-OHF**				
Kishida and Fukushima, 1977	6	NA	530 ± 156 ng/ml, range 313–683 ng/ml	Urine spot sample
Pal, 1980	20	2–7	41±7 µg/24h, range 30–52 µg/24h	24 h urine collection
Patel et al., 1996	12 (term)	15–90	31.67 ± 6.55 ng/ml	4 h urine sample
**6*β*-OHF/cortisol ratio**				
Vauezelle-Kervroudan et al., 1996	7 (preterm) 13 (term)	1–15	7.2 ± 3.8 (preterm) 7.9 ± 5.6 (term)	Urine spot sample
Nakamura et al., 1998	42 (preterm) 39 (term)	1	5.3 ± 0.9 (preterm) 16.5 ± 1.9 (term)	Urine spot sample
Nakamura et al., 1999	56 (term)	1 3 5 7	17.6 ± 7.8 10.9 ± 7.1 8.7 ± 5.5 6.8 ± 3.5	Urine spot sample
**CPI en CPIII**				
Wolkoff and Arias, 1974	23 (preterm)	NA	11.2 ± 8.4 µg/m²/24 u (CPI) 6.3 ± 5.3 µg/m³/24 h (CPIII)	Random urine samples
Minder and Schneider-Yin, 1996	18	4-10	17.08 (35.68) µmol/mol creatinine (CPI)^b^ 8.65 (22.75) µmol/mol creatinine (CPIII)^b^	Random urine samples
Ozalla et al., 2002	68 (term)	3	13.7 (6.3–18.7) (CPI)^c^ 10.2 (5.2–20.5) (CPIII)^c^	Spot urine sample
Kunitata et al., 2016	31 (15 preterm)	0–6 months	≥0.3 in 80% of infants [CPI / (CPI + CPIII)]	Urine spot sample

Values assessed in pathologic conditions as well as values collected in the same subjects but after the neonatal period, were excluded. ^a^Values are expressed in median (range) or mean ± SD, unless otherwise stated; ^b^values expressed as mean (mean+2SD); ^c^values expressed as total population median and QR. Population consisted of 38 cases with maternal exposure to airborne hexachlorobenzene and 30 cases without exposure; QR, quartile range; PNA, postnatal age; NA, not available; 4β-OHC, 4beta-hydroxycholesterol; 6β-OHF, 6beta-hydroxycortisol.

##### 4*β*-OHC

Is exclusively formed from cholesterol in the liver and gut by CYP3A4, with only a minor contribution from CYP3A5. 4*β*-OHC displays low intraindividual variability (7.1% range 4.8–13.2% over 3 months, and a −8.2 to 1.7% change from baseline over a period of 3–30 days in healthy volunteers) and has a long half-life (60 h to 17 days in adults) with CYP7A1, the rate limiting enzyme in bile acid synthesis, as considered responsible for the slow elimination of 4*β*-OHC (Mao et al., 2017). Therefore, 4*β*-OHC represents a weighted average of CYP3A activity over a period of several days (Tomalik-Scharte et al., 2009). This allows the quantification of baseline CYP3A activity from a single blood sample. The impacts of genetics, gender, and to a limited extent, disease as factors contributing to interindividual variability in 4*β*-OHC have been investigated in adults (Mao et al., 2017). Recently, CYP3A activity was observed to be lower in type 2 diabetic compared to nondiabetic subjects, and this decrease was reflected in 4beta-OHC concentrations and 4beta-OHC/cholesterol ratio (Gravel et al., 2019). Based on 4*β*-OHC plasma concentrations in Swedes, Koreans, and Tanzanians, Diczfalusy et al. reported interethnic differences in both CYP3A4 activity and the frequency of active CYP3A5*1 alleles (Diczfalusy et al., 2011). In addition, the authors reported that women have higher 4*β*-OHC concentrations compared to men. Also, in Ethiopians, higher 4*β*-OHC and 4*β*-OHC/cholesterol ratio was documented in women compared to men (Gebeyehu et al., 2011).

Overall, the relationship between CYP3A activity and 4*β*-OHC is complex, and there are different opinions on the role and validity of 4*β*-OHC as biomarker. Based on a study in healthy adults and Human-Immunodeficiency Virus positive patients, Tomalik-Scharte et al. showed only a weak correlation of 4*β*-OHC and midazolam (as prototypic CYP3A substrate) clearance. The authors concluded that the currently available data do not justify the use of 4*β*-OHC as endogenous CYP3A biomarker, especially not for assessing basal CYP3A activity. Only for changes in CYP3A activity driven by strong inducers and inhibitors it may be useful (Tomalik-Scharte et al., 2009). Kasichayanula et al. reported that changes in 4*β*-OHC are useful as surrogate for midazolam PK after multiple doses of a potent CYP3A inducer (Kasichayanula et al., 2014). However, since the relationships found between 4*β*-OHC and midazolam CL in different reports have statistical limiations, Ma et al. do not believe that 4*β*-OHC/cholesterol ratio has been validated as a biomarker for CYP3A activity (Ma et al., 2013). In addition, reports on poor correlations with the clearance of CYP3A substrates *e.g.* midazolam are available (Neuhoff and Tucker, 2018). To further illustrate this, currently available clinical data and simulation analyses support the use of 4*β*-OHC to assess potential CYP3A inducers in drug development (Kasichayanula et al., 2014), but the change in 4*β*-OHC concentration is often not linear to the change in specific compound clearance. The same holds true for CYP3A inhibitors, since no general trend between the responses of 4*β*-OHC in the presence of CYP3A inhibitors can be seen. Based on some of the limitations mentioned above, it was recently questioned if 4*β*-OHC would ever be a robust quantitative biomarker of CYP3A4 activity. In addition, after formation of 4*β*-OHC, it is further metabolized by CYP7A1 and CYP27A1. The contributions of all CYP isoforms involved finally determine the 4*β*-OHC plasma concentration (Neuhoff and Tucker, 2018). In conclusion, although this biomarker and its ratio with cholesterol are considered as promising in adults, further research and validation studies are needed. Due to its long half-live, 4*β*-OHC is not appropriate to evaluate rapid changes in CYP3A activity (Mao et al., 2017). Exogenous markers like midazolam or quinine are preferable for short-term studies, while 4*β*-OHC is a marker of CYP3A activity in long-term studies, especially to assess induction, but also inhibition (Diczfalusy et al., 2011). At present, 4*β-*OHC is mainly used in research settings. Mao et al. hereby recommend an investigation of hepatic CYP3A activity in clinical pharmacology studies using 4*β*-OHC (Mao et al., 2017). In addition, these authors state that assessment of the baseline 4*β*-OHC concentrations in special populations, *e.g.* children, is one of the current knowledge gaps in 4*β*-OHC.

Observations in neonates are limited. Nylen et al. determined 4*β*-OHC and 4*β*-OHC/cholesterol ratio at birth and 4 months PNA in 21 neonates (Nylen et al., 2011). At birth, neonates had lower plasma 4*β*-OHC than their mother as well as healthy adults. This was attributed to the low cholesterol values in newborns, which increased fast and even more than doubled in the first 4 months of life. Neonates had a median ratio (0.19) comparable to adults already at birth (Nylen et al., 2011). This ratio did not change during the first 4 months of life. The different CYP3A activity ontogeny profiles reported based on 4*β*-OHC or probe compounds (Stevens et al., 2003; Johnson et al., 2008) may be related to the different substrate specificities of CYP3A enzymes. It is also not clear if CYP3A7 (fetal expression) would actively contribute to 4*β*-OHC formation.

##### 6β-OHF

Cortisol is metabolized to 6*β*-hydroxycortisol by CYP3A4, mainly in the liver and also in the kidney, placenta, and adrenal glands, and it is excreted in urine. In line with the findings on 4*β*-OHC, conflicting evidence can also be found on the relevance of 6*β*-OHF as endogenous CYP3A biomarker. Based on a review of 277 papers, Galteau and Shamsa conclude that urinary 6*β*-OHF is a useful parameter to evaluate enzyme inducing or inhibiting characteristics of drugs in case the subjects are their own controls, due to the extensive interindividual variability in cortisol metabolism. They consider 6*β*-OHF excretion not reliable enough to determine actual CYP3A4 activity (Galteau and Shamsa, 2003). Interindividual variability in 6*β*-OHF is in part attributed to variability in CYP3A4 expression. Studies investigating the impact of gender on 6*β*-OHF concentrations, both in adults and in children, are contradicting, but mostly not statistically significant (Galteau and Shamsa, 2003; Eldesoky et al., 2007). Since cortisol itself displays a diurnal variation, and intraindividual variability in 6*β*-OHF concentrations differ across reports, the 6*β*-OHF/cortisol ratio is often suggested to be more useful. According to Chen et al., the intraindividual variability of this ratio is moderate, and, measured in a single urine sample, this ratio reflects reasonably well the mean value of the ratio over a period of 2 months (Chen et al., 2004). In contrast, another group compared total body clearance of midazolam with the urinary 6*β*-OHF/cortisol ratio as markers of CYP3A activity but was unable to demonstrate a significant correlation (Chen et al., 2006). Although midazolam and cortisol both are Class 1 BCS compounds, the high intra- and interindividual variability in the cortisol ratio may mask the possible correlation between both biomarkers. These authors therefore consider the ratio as a suboptimal phenotyping tool for CYP3A (Chen et al., 2006). On the other hand, Shin et al. reported that the 6*β*-OHF/cortisol ratio is the best predictor for hepatic CYP3A activity under both maximal inhibition and maximal induction and concluded that a predictive model including this ratio as covariate is useful to predict the impact of drug interactions mediated by CYP3A (Shin et al., 2016).

In neonates, reports on urinary 6*β*-OHF concentrations are highly variable, from high (530 +/− 156 ng/ml) (Kishida and Fukushima, 1977), to very low values (31.67 +/− 6.55 ng/ml) (Patel et al., 1996). For 6*β*-OHF/cortisol ratio, also conflicting results can be found. Vauzelle-Kervroudan et al. reported higher ratios in preterm and term neonates (PNA 1–15 days) compared to older infants (30–359 days) (Vauzelle-Kervroedan et al., 1996), while others found different or opposite results (Nakamura et al., 1998). Nakamura et al. found a correlation between the ratio and GA and also birth weight (Nakamura et al., 1998). For preterm neonates, the ratio at birth was lower compared to term cases and remained stable the first 14 days of life (Nakamura et al., 1998). However, the same group reported that 6*β*-OHF/cortisol ratio in term neonates was high on the day of birth, independent of the CYP3A activity of their mothers. The neonatal ratio subsequently decreased after birth in these term cases and reached a level equal to preterm cases at PNA 5 days. Although a full explanation is not yet available, alterations of CYP3A7 in neonates are suggested as a possible contributing factor (Nakamura et al., 1999). In young children, a different evolution of 6*β*-OHF at PNA day 90 *versus* 195 was seen, depending on if and which medication their mother was taking during breastfeeding (Toddywalla et al., 1995). Overall, in adults, 6*β*-OHF/cortisol excretion decreases after the age of 50–60 years. Results in neonates and children on this trend are conflicting and need further research (Galteau and Shamsa, 2003).

##### CP1 and CPIII

Both *in vitro* and *in vivo* studies indicate the suitability of coproporphyrins (CPs) as endogenous biomarkers for (OATP)1B-mediated drug–drug interactions. The human (OATP) 1B1 and 1B3 are DT, which are expressed at the sinusoidal membrane of hepatocytes (Kunze et al., 2018). CPI and CPIII are byproducts of the heme biosynthesis and are eliminated by hepatobiliary and renal route. CPI and CPIII are substrates of OATP1B1 and 1B3. In addition, CP1 is substrate of multidrug resistance-associated protein (MRP) 2, while CPIII is also defined as a substrate of OATP2B1 (Kunze et al., 2018). Kunze et al. observed that changes in plasma concentrations of these markers due to mild to strong OATP1B inhibitors were predictive for moderate and strong OATP1B-mediated drug–drug interactions and that CPI exposure was useful to distinguish between mild and moderate interactions (Kunze et al., 2018).

In the newborn period, CPI is the predominant fraction of excreted CPs, *i.e.* about 68.4 ± 12.3% of total CPs. Subsequently, CPI concentration and its fraction of total CP decrease in the first 6 months of life and approach the adult values at age 9 years (Minder and Schneider-Yin, 1996). Excretion of CPI occurs by biliary-fecal route, in line with bilirubin. This elimination pathway is still immature in neonates. Minder and Schneider-Yin suggested that the excess of CPI, although hydrophobic, is forced into the renal elimination route due to an overloaded biliary-fecal transport (Minder and Schneider-Yin, 1996). The predominance of CPI in neonates is also documented for preterm cases. Based on urinary data, preterms excrete a significantly higher percentage of total CP as CPI isomer (59.4 ± 17.3%) compared to healthy adults (24.6 ± 5.6%). This CPI excretion (expressed in µg/m²/24 h) was not significantly different in preterms compared to healthy adults. CPIII urinary excretion (expressed in µg/m²/24 h) was significantly lower in preterm neonates compared to healthy adults (Wolkoff and Arias, 1974). Recently, Kunikata et al. investigated the developmental pattern of urinary CP[I/(I+III)], as a marker for the ATP-binding cassette, subfamily C, member 2 (ABCC2) function. The authors observed that the CP[I/(I+III)] ratio varies extensively in infants up to 6 months of age. Furthermore, the ratio is inversely correlated with corrected GA, and lowest values are achieved at the age of 1–2 years, suggesting highest ABCC2 activity at that age. In summary, although the currently available data are promising, additional research is needed to further unravel the applicability and selectivity of CPI and CPIII as endogenous biomarkers.

#### *In Vitro* Approaches to Explore the Impact of TH on PK in Neonates

As compared to the significant insights regarding the impact of hypothermia on renal drug elimination (see **Table 1**), quantitative knowledge regarding the impact of TH on the function of DME and DT is much more limited. Nevertheless, very few *in vitro* studies have specifically explored the impact of moderate hypothermia (32–34°C) on the activity of DME. For instance, cytochrome P450 (CYP)-mediated ethyl-morphine *N*-demethylation in pig liver microsomes was about 31% lower at 32 *versus* 38°C (Fritz et al., 2005). A few more studies have reported the effects of severe hypothermia (26°C) on drug metabolism, mostly in model systems prepared from animals (Van Den Broek et al., 2010). For instance, McAllister et al. (Mcallister and Tan, 1980) demonstrated that the affinity (=1/Km) of verapamil and propranolol for DME was decreased significantly at 26°C compared to 37°C. This observation also implies significantly reduced metabolizing capacity at (substantially) reduced temperatures.

Given the predominant role of the liver in metabolic drug elimination, future *in vitro* research in this context should focus on the full exploitation of multiple liver-derived *in vitro* models for drug elimination, *e.g.* primary hepatocytes in different configurations (suspensions, cultures), microsomes, or recombinantly expressed cytochrome P450 (CYP) enzymes. The combined use of multiple *in vitro* models carries the possible advantage that intrinsic effects of temperature on DMET that are also present in extrahepatic locations (*e.g.* the intestine) may be derived.

The major advantages of *in vitro* tools to explore the temperature dependence of hepatic drug metabolism are: (i) experimental conditions, in particular temperature, can be strictly controlled; (ii) the temperature effect on biotransformation processes can be evaluated for a sufficiently broad range and at high resolution (*i.e.* with high precision); (iii) the primary underlying mechanisms (*e.g.* transporters *versus* drug metabolizing enzymes) of the possible temperature dependency can be evaluated; (iv) by selecting the proper *in vitro* tools immediate *versus* delayed effects of temperature can be evaluated; (v) in view of the significant throughput of *in vitro* tools for hepatic drug disposition, multiple compounds can be studied, enabling to evaluate the genericity *versus* drug-specificity of the findings; (vi) the use of pooled hepatocytes *versus* hepatocytes from individual donors allows for exploring the possible intersubject variability of the temperature-dependent processes. Correlation of donor-specific effects with donor demographics may also support implementation of donor-specific factors (*i.e.* explaining part of the intersubject variability) to the eventual PBPK framework. Specifically, in the context of TH in neonates, the influence of temperature on hepatic drug disposition processes can be studied in hepatocytes obtained from neonatal *versus* adult liver tissue donors. At the same time, it should be noted that heterogeneity in available batches of neonatal hepatocytes will also present the challenge of unknown effects of numerous other covariates.

Specifically, to explore the effect of TH on hepatic drug disposition proteins, it is important to distinguish between immediate (4–6 h) and delayed (> 24h) effects of hypothermia. Immediate effects encompass the influence of temperature on protein stability (*i.e.* degradation rate) as well as the effect of temperature on catalytic protein activity (*i.e.* intrinsic activity). Delayed effects are considered the result of the possible influence of temperature on protein expression. Such effects may include mechanisms of altered activation of nuclear receptor-mediated pathways, as well as the possible influence of temperature on transcriptional and translational activities. Proposed methodological details for evaluating both immediate and delayed effects of temperature on hepatic DME and DT have been summarized in **Table 3**.

**Table 3:** Proposed experimental conditions for *in vitro* evaluation of the immediate *versus* delayed effects of hypothermia on activity of drug metabolizing enzymes (DME) and drug transporters (DT).

Immediate effects of hypothermia on *in vitro* activity of DME and DT
*Model systems and selected DME and DT* Both pooled human liver microsomes, S9 fractions and suspended human hepatocytes (2 pooled and at least 3 individual neonatal donors) should be used as model systems to evaluate the impact of hypothermia (33–37°C) on the activity of the most important hepatic DME, including CYP1A2, CYP2B6, CYP2C8/9/19, CYP2D6, CYP3A4, CYP2E1, Flavin- Containing Monooxygenase (FMO)1-3, uridine 5 ´-diphospho-glucuronosyltransferases (UGT's), aldehyde oxidases, and esterases; Suspended human hepatocytes (pooled and individual donors) should be used to determine the effect of hypothermia on the major drug uptake transporters [OATP1B1/3, sodium-taurocholate cotransporting polypeptide (NTCP), organic cation transporter (OCT)1, organic anion transporter (OAT)2] and the sinusoidal efflux transporter MRP3 (multidrug resistance-associated protein 3); Sandwich-cultured human hepatocytes (from at least 3 individual donors) and membrane vesicles to determine the effect of hypothermia on activity of key canalicular [MultiDrug Resistance protein (MDR)-1, Multidrug Resistance-associated Protein (MRP)-2, Breast Cancer Resistance Protein (BCRP)] efflux transporters; Similar *in vitro* experiments can be designed in at least 2 batches of minipig hepatocytes. Experiments with animal hepatocytes support unique insights into *in vitro in vivo* extrapolation (IVIVE) provided corresponding in *vivo* studies in the same species are also conducted.
*Study design and model compounds* To allow distinction between effects on protein stability *versus* catalytic activity, incubations with the *in vitro* model systems should be conducted either during hypothermia, or immediately after an *in vitro* hypothermia episode (60 min). Multiple cycles of hypothermia and warming may also be considered in an attempt to mimic the *in vivo* impact of hypothermia (for 72 h) and warming on DME and DT activity. Depending on the research question, a strategic selection of probe substrates should include: ...the hepatically metabolized model compounds of interest (*e.g.* midazolam, fentanyl, phenobarbital because of their clinical relevance in the neonatal population); ...established compounds for *in vitro* DME and DT phenotyping, and; ...compounds used as *in vivo* biomarkers of specific DME and DT activity, thus enabling more straightforward data interpretation as a well as scaling towards the *in vivo* context.
*Current challenges* Relatively high inter-donor and/or inter-occasion variability in the obtained *in vitro* data is to be expected. This may complicate the use of the data for a robust PBPK model. Mitigation strategies include more emphasis on the use of pooled microsomes/hepatocytes as well as the use of *in vitro* model systems expressing single heterologously expressed DME and DT. The relatively large number of conditions to be evaluated necessities a tiered approach, based on initial selections of DMET model systems and substrates.
**Delayed effects of hypothermia on mRNA expression, protein abundance and activity of hepatic DME and DT**
*Model systems and selected DME and DT* Sandwich-cultured human (and possibly animal) hepatocytes (from at least 3 individual donors) should be used to determine the delayed (>24 h after hypothermia episode) effect of hypothermia (72 h) on the mRNA expression and protein abundance of key DME (CYP1A2, CYP2C9, CY3A4) and DT (OATP1B1/3, MRP).
*Study design and model compounds* Sandwich-cultured hepatocytes offer the advantage that hepatocytes can be used in study designs covering more than 1 week. The activity assays described for studying immediate effects (see above) are also applicable in this model system.
*Current challenges* Altered protein expression and activity in cultured hepatocytes will complicate extrapolation to *in vivo* effects. Analogous to interpretation and extrapolation of *in vitro* induction response in cultured hepatocytes (Zhang et al., 2014), parallel evaluation of compounds exhibiting known *in vivo* impact of T° will be required.

#### *In Vivo* Studies in Animal Models

Much of the current understanding of PA in man has been derived from animal studies. As pointed out in a review by Michael Painter, the use of animal models for this condition has a long history (Painter, 1995). Resistance of the neonate to asphyxia, in terms of duration of survival, has already been described in the early days in several animal models that were submitted to different ways of asphyxial insult (Fazekas et al., 1941; Glass et al., 1944; Britton and Kline, 1945; Stafford and Weatherall, 1960). There is a good correlation between maturity and resistance to asphyxia. Males appear to be less resistant than females (Britton and Kline, 1945), and high body temperature also reduces resistance. In neonatal rats for example, it has been clearly shown that lowering the body temperature from 37 to 30°C during asphyxia has a protective effect (Saeed et al., 1993). Altricial species are more resistant to asphyxia than precocial species, and asphyxia has been studied in a wide variety of animal models, including cats, dogs, guinea pigs, sheep, nonhuman primates, pigs, rabbits, rats, and even bats (Painter, 1995). Mice and rats have been shown to be useful models to study the biochemical and pathological changes in the gray and white matter of the brain following perinatal asphyxia, but they are not considered to be appropriate models for studying damage to peripheral organs such as the kidney (Larosa et al., 2017). Large precocial animal models are much more useful in this regard as they more closely mimic the human pathophysiological response to PA, although they also have disadvantages when considering their life span and consequently the life course of *e.g.* hypoxic–ischemic encephalopathy.

Primates are definitely closer to humans on the phylogenetic tree, but this does not always mean they are more suitable asphyxia models. Body composition and hormonal changes during pregnancy differ between man and nonhuman primates. The nonhuman primate is much more advanced at birth than the term human regarding its adaptive and neuromotor maturity (Painter, 1995). Despite these limitations, nonhuman primate studies have clearly shown that the immature fetal brain has a greater degree of tolerance to an asphyxial insult than the mature brain, and ischemia is also required in addition to a reduced PaO_2_ to cause measurable brain damage (Raju, 1992).

Due to the low cost and large size, the lamb has extensively been studied under asphyxia conditions. It has been shown to be a very valuable model to study cerebral blood flow and metabolism (as reviewed by Raju, 1992). However, the placental flow pattern and cerebral vascular anatomy are distinctly different from those in man. Furthermore, it is unclear whether the neuropathological changes in the lamb are comparable to those in humans.

The neonatal piglet is also a good model to study the cerebral blood flow and metabolism, and the model has also been well-standardized in conventional pigs. It provides several advantages such as similar development as that of a 36 to 38 weeks old human fetus with comparable body systems, large body size at birth (1.5–2 kg) that allows the instrumentation and monitoring of the animal. Furthermore, the confounding variables of hypoxia and hemodynamic derangements can be controlled (Cheung et al., 2011). As the piglet asphyxia model is so well-characterized, this also opens opportunities for pharmacologic interventions and procedures applied in a clinical setting, such as TH. For renal drug excretion, this has already been shown with gentamicin. GFR in neonates with PA was about half the GFR of neonates without PA, resulting in a decreased CL of gentamicin. In addition, hypoxia–ischemia-induced renal impairment itself also delays the excretion of gentamicin (Satas et al., 2000). However, the impact of TH on hepatic metabolism of administered drugs remains unclear and is difficult to study as a separate covariate. As such, the piglet asphyxia model could provide very useful information. The PK of dexmedetomidine combined with TH has been studied in a piglet asphyxia model by Ezzati et al., (2014). These authors showed that dexmedetomidine clearance was reduced almost 10-fold in the newborn piglet following hypoxic–ischemic brain injury and subsequent TH compared with adult values. These high plasma levels of the drug were associated with major cardiovascular complications. However, the exact contribution of hypothermia to the decreased clearance was not investigated in this study.

Furthermore, in view of new medicines under development for the pediatric population, the conventional pig is not used. The Göttingen Minipig is the most commonly used pig strain in nonclinical drug development in Europe, as is the Beagle when using the dog and the Sprague Dawley or Wistar when using the rat. The Göttingen Minipig has several advantages compared to conventional pigs, such as its small size, and they are easy to handle, but the main advantage is that they are genetically coherent. As such, they are well-characterized, and by using the same strain, pharmaceutical companies can rely on historical control data when interpreting findings in the safety studies. At this point, drug metabolism has already been studied in Göttingen Minipigs from fetal stage up to weaning, which corresponds to approximately a 2-year old child, and in adults (Van Peer et al., 2014; Van Peer et al., 2017). These studies showed that the Göttingen Minipig has in general a similar ontogeny pattern of CYP- and UGT-mediated metabolism compared with neonates up to 2-year old children. This strengthens the expectation that establishing an asphyxia model with TH in this porcine strain could provide useful information for the human condition.

In conclusion, animal models of PA may not entirely represent the human situation, but it is clear that they have advanced the understanding of this disease in man and still provide new opportunities. In this regard, a neonatal Göttingen Minipig asphyxia model with TH could provide very useful data for drugs under development for pediatric use, but also for drugs that are already currently used in neonatal intensive care units.

### Strategy for PBPK Framework Evaluation

#### PBPK Hypothermia Framework Evaluation Using *In Vivo* Animal and Human Data

As mentioned before, human (healthy) neonatal PBPK models are already commercially available, and also for the adult (Suenderhauf and Parrott, 2013) and juvenile Göttingen Minipig (Van Peer et al., 2015) steps have been undertaken to build PBPK models in this species. However, datasets on *in vivo* drug disposition during asphyxia and/or hypothermia in the neonatal Göttingen minipig and human neonatal cohorts need to be built and can then be used to further construct and evaluate the PBPK models. So far, only the combined impact of asphyxia and hypothermia on disposition of drugs undergoing renal elimination without metabolism has been well covered in man and piglets. In contrast, *in vivo* disposition of drugs requiring metabolism under hypothermia conditions is a knowledge gap. As the potential role of hypothermia and asphyxia on drug disposition in neonates cannot be investigated as separate covariates in the clinical situation, a porcine neonatal *in vivo* model can provide very useful data. More specifically, the following four conditions can be investigated *i.e.* first, the clinical situation by administering compounds, selected based on their different physicochemical/PK characteristics and clinical relevance, such as midazolam [CYP3A4; intermediate extraction ratio (ER)], phenobarbital (CYP2C19; low ER), topiramate (largely renally excreted unchanged), and fentanyl (CYP3A4; high ER), in asphyxiated neonatal Göttingen minipigs during TH. A second group contains asphyxiated neonatal piglets under normal body-temperature, whereas a third group contains nonasphyxiated neonatal piglets during hypothermia. Controls, *i.e.* nonasphyxiated, normothermic piglets, also need to be included. For human neonates with PA combined with TH who often receive multiple drugs, available published datasets on human *in vivo* observations of drug disposition can be used to evaluate and further improve the PBPK hypothermia framework.

#### PBPK Software Qualification

According to the EMA guideline (EMA/CHMP/458101/2016—effective mid 2019) on the reporting of PBPK modeling and simulation, PBPK modeling efforts supporting regulatory submissions require qualification of the software platform for the intended use. The guideline lists expectations on how to qualify such a software platform. In addition, demonstration of predictive performance of PBPK models is considered an essential part of PBPK M&S reports. It goes without saying that such recommendations are also highly relevant in the context of the development of a PBPK platform for dose prediction in neonates under TH. Importantly, the guideline specifies that PBPK models designed to predict pediatric drug dosing are considered to be high (regulatory) impact applications.

Apart from model verification in terms of mathematical structure, especially the availability of clinical reference, data is crucial to support platform qualification. The guideline states that several compounds with comparable ADME characteristics should be included in a qualification ‘package'. It is not surprising that inclusion of a higher number of ‘qualification compounds' will generally lead to higher confidence in the PBPK platform. The selection of such compounds will depend on the availability of *in vitro* and *in vivo* data, as well as on the contribution to the data set in terms of diversity of PK and physicochemical characteristics. For the hypothermia PBPK models, we provided model compound suggestions in the section *PBPK Hypothermia Framework Evaluation Using In Vivo Animal and Human Data*.

As already mentioned, this guideline also provides a framework for evaluation of the predictive performance of the drug-specific PBPK models. This essentially implies testing the performance of the model under numerous different conditions such as for example: (i) different populations; (ii) single *versus* multiple dosing; (iii) dose-dependency (or lack thereof); (iv) different dosage forms and/or routes of administration; (v) supportive evidence from a corresponding nonclinical PBPK model. It is noted that simulations should include a sufficiently large number of subjects, which may help to predict population variability of the outcomes.

Last but not least, developed PBPK models should be subjected to sensitivity analyses, providing insight into the impact of imprecision in input variables (*e.g.* Log P, intrinsic hepatic clearance) on predicted drug exposure. Plausibility regarding the underlying drug disposition mechanisms can provide guidance regarding the relevance to analyze the sensitivity towards particular input variables.

## Discussion

PBPK modeling and simulation can provide a link in translating *in vitro* observations to real *in vivo* impact. It supports the decision making about the need and priority of clinical trials, but also for drug dose selection for a special patient population. The strategy for the development and evaluation of a neonatal hypothermia PBPK model, provided in this review, can be used for personalized drug therapy and drug development. In the next step, we aim to illustrate how this strategy can contribute to achieve model-informed precision dosing in neonates with PA undergoing TH.

### Case Example With Midazolam as Model Drug

To provide an initial illustration regarding the potential feasibility (and limitations) of PBPK modeling to predict the impact of TH on PK, midazolam was chosen as a model drug based on: (i) the availability of a significant amount of clinical PK data, also in neonates; (ii) its PK profile, in view of the fact that it is an intermediate ER drug. The latter implies that changes either in hepatic blood flow or changes in intrinsic hepatic CL may affect its PK behavior.

The clinical data on midazolam PK in 19 neonates on extra-corporeal membrane oxygenation as published by Mulla et al. (Mulla et al., 2003) were used as reference *in vivo* data because: (i) newly generated simulations following iv infusion can be directly compared to simulations published as part of the Mulla study; (ii) ECMO-independent reference values for both CL and Vd at steady state had been published; (iii) demographics of the population including body weight (BW, mean 3.4 kg) are available.

**Figure 4:**
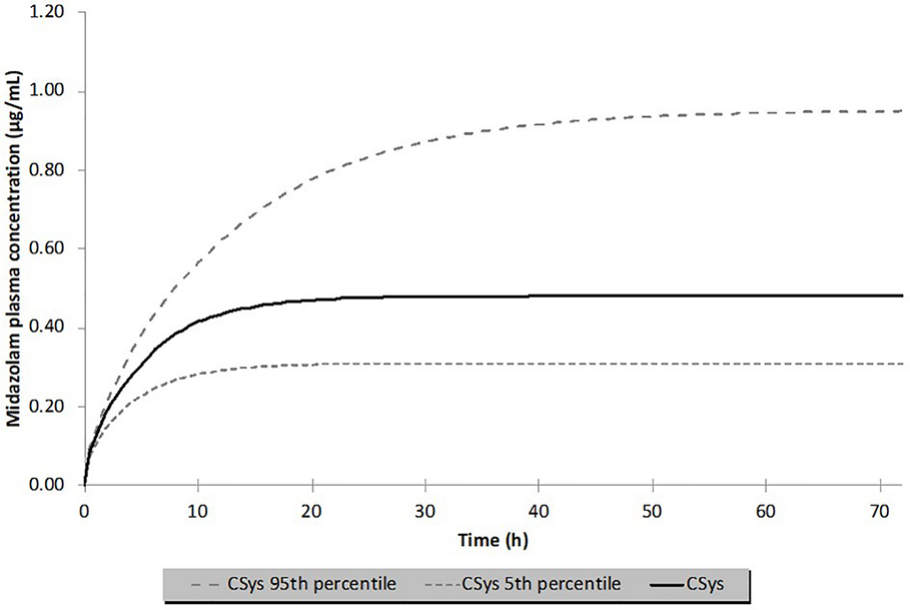
Median (± 90% CI) predicted systemic concentrations (C_sys_) of midazolam following intravenous infusion at 0.05 mg/h/kg in 19 normothermic neonates (mean body weight 3.91 ± 0.86 kg) for 72 h (total dose 3.6 mg/kg). The solid line and the dashed lines represent the median and 5/95% percentiles. The median concentration at steady state was 0.48 µg/ml.

The PBPK models described below were all developed in Simcyp v.18 (Certara) using the default physiochemical parameters for midazolam and the default pediatric population, selecting an age range of 0–28 days (neonates). Initial model development included selection of the ‘full PBPK'-mode, using a modified scalar on the tissue/plasma partition coefficient (Kp) values in order to satisfy the Vd of 0.82 L/kg as reported by Mulla et al. However, as opposed to the simulation published by Mulla et al. and for reasons of simplicity, no loading dose was administered, but the same maintenance dose level of 0.05 mg/h/kg BW was given for 72 h (total 3.6 mg/kg), which is the typical duration of the TH. **Figure 4** illustrates the median (± 90% CI) predicted systemic concentration time profile for midazolam in a virtual population of 19 ‘Simcyp neonates' (mean BW 3.9 kg). The steady-state concentration (C_ss_)of 0.48 µg/ml is about 20% lower than the C_ss_ published by Mulla et al. (~0.6 ~µ/ml). However, comparison of an individually predicted concentration–time (C–t) profile for a BW of 3.4 kg (corresponding to the median BW in Mulla et al.), reveals a C_ss_ of 0.74 µg/ml, providing confidence in the predictive performance of the model, even though there appears to be a significant impact of BW on hepatic CL. One can expect that careful matching of virtual patients with the patients in the reference study will further improve the predictive performance.

**Figure 5:**
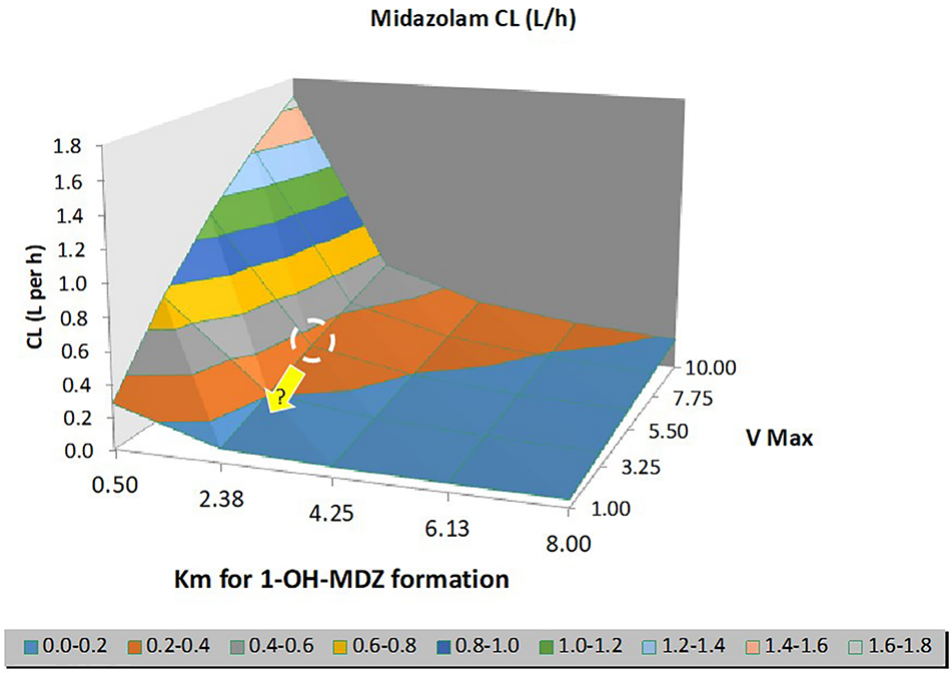
Output of a sensitivity analysis for midazolam clearance (CL) in neonates, illustrating the model-predicted impact of changes in Michaelis–Menten constant (K_m_) and/or maximum reaction rate (V_max_) describing the intrinsic formation CL of 1-OH midazolam by CYP3A4. The ‘dashed' circle represents the V_max_/K_m_ values at normothermia, while the yellow arrow indicates a plausible change in V_max_ (rather than K_m_) under conditions of hypothermia.

**Figure 6:**
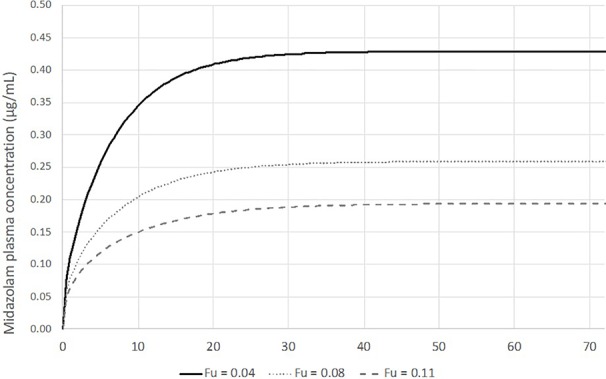
Output of a sensitivity analysis for midazolam clearance (CL) in neonates, illustrating the model-predicted impact of changes in unbound fraction in plasma (fu_plasma) assuming a fixed blood/plasma ratio (B/P ratio) of 0.55. A sensitivity analysis for the B/P ratio between 0.55 and 1.2 did not reveal any significant changes in hepatic CL. During normothermia, the reported fu value is 0.04.

**Figure 7:**
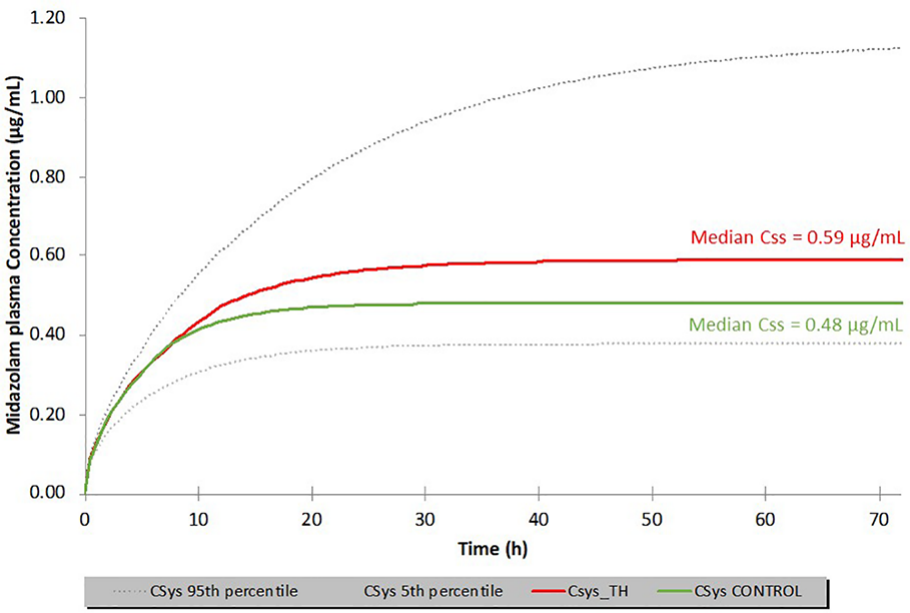
Simulated midazolam median systemic plasma concentrations (C_sys_) in neonates (n = 19; 0.05 mg/h/kg for 72 h; total dose 3.6 mg/kg) under normothermic (green line) *versus* hypothermic (red line) conditions. Therapeutic hypothermia (TH) was assumed to induce a 20% change in each of the following parameters: cardiac output (decreased), unbound fraction (fu, increased), blood/plasma ratio (B/P ratio, decreased) and 1-OH-midazolam formation intrinsic clearance (CL, decreased). Dashed lines represent the 90% CI for the hypothermic condition. C_ss_: steady state concentration.

With this ‘basic' neonatal PBPK model for iv midazolam, several sensitivity analyses were conducted to evaluate the possible impact of TH on midazolam PK. Anticipated temperature-induced alterations were explored for the following parameters: (i) intrinsic hepatic formation CL for 1-OH-midazolam (CYP3A4) (**Figure 5**); (ii) 20% reduction in cardiac output (CO); (iii) changes in blood/plasma ratio (B/P ratio) and/or unbound fraction in plasma (fu_plasma) (**Figure 6**). As shown in **Figures 5** and **6**, realistic changes in Michaelis–Menten constant (K_m_) and/or maximum reaction rate (V_max_) for the 1-OH midazolam formation and in fu_plasma (rather than B/P ratio) appeared to have a potentially clinically significant impact on *in vivo* (hepatic) CL of midazolam. On the other hand, a 20% reduction in CO did not have a substantial impact on the predicted CL (data not shown). Also, the influence of changes in renal midazolam CL were evaluated but no effects were seen, more than likely due to the limited contribution of renal CL to overall midazolam CL. In order to construct a more realistic scenario, a hypothetical impact (*i.e.* 20%) of reduced temperature on a combination of multiple parameters (CL_int_, CO, B/P ratio and fu_plasma) was subsequently simulated (see **Figure 7**). A 23% increase in the median C_ss_ of midazolam was predicted based on these assumed changes in parameters under TH. This also implies a 20% decreased CL as C_ss_ solely depends on the infusion rate and CL.

While additional *in vitro* data on intrinsic metabolism as well as *in vivo* physiological data (including the impact of moderate hypothermia) are needed, this initial set of simulations suggests the utility of PBPK modeling to interrogate the impact of TH regimens in neonates on PK. Clearly the predictive performance of such models will highly depend on the scope and richness of available input data. For instance, Empey et al. (Empey et al., 2012) have previously shown that primarily metabolic capacity (rather than enzyme affinity) affected systemic clearances of fentanyl and midazolam. This finding primarily guides towards evaluating temperature-dependent changes in V_max_ (but with constant K_m_) on simulated midazolam levels in human neonates (see **Figure 5**).

These simulations are clinically relevant since they improve our understanding of compound-specific disposition in a well-defined pathophysiological condition and can be used to derive model-informed precision dosing. Beyond the compound-specific knowledge, these data are useful to further explore the impact of other nonmaturational factors *e.g.* drug–drug interactions (Allegaert et al., 2019). Based on *in vivo* observations, co-administration of phenobarbital, a CYP3A inducer, will for example increase midazolam CL in neonates treated with TH with a factor 2.3 (95% CI 1.9–2.9) (Favie et al., 2019). This knowledge allows for continuously refining available PBPK models.

### Reflections and Future Directions of Temperature-Driven PBPK Models

Below, some additional reflections on the need, applicability, and future directions of a PBPK hypothermia framework are made. At present, TH has an effect size of 15% in the Western world. However, in low- and middle-income countries where low-cost hypothermia techniques are applied, no significant reduction in neonatal mortality is documented (Pauliah et al., 2013; Albrecht et al., 2019). Preventive measures to limit oxidative stress-related brain injury in neonates with PA is especially important in low-income countries since poor prenatal care and limited access to health care contribute to neonatal morbidity and mortality. Early obstetrical interventions as well as tailoring neuroprotective strategies to settings limited to resources and technology are needed (Solevag et al., 2019). Adequate randomized controlled trials in this setting are challenging but needed before TH can be offered as standard of care. Albrecht et al. also add that alternative rescue treatments, including neuroprotective pharmacotherapy, need to be cheap and easy to administer. In addition, therapy needs to remain stable and not affected by ‘high' environmental temperature. On the one hand, this nicely fits in the ‘asphyxia' part of our PBPK framework. On the other hand, this also illustrates the broader need for temperature-driven (high as well as low temperature) PBPK models.

The PBPK framework presented in this review is beneficial for both academic and industry-driven research since new data generation may facilitate development of temperature-driven PBPK models for other populations or for other indications. As highlighted by Gancia and Pomero, hypothermia is a promising future therapy for acute ischemic stroke and improves survival and outcome in adults with cardiac arrest. Consequently, further research on other cooling modalities (timing, duration) and indications (*e.g.* preterms, perinatal ischemic stroke, postresuscitation, necrotising enterocolitis) in neonates and also in other patient populations is suggested (Gancia and Pomero, 2011). Within the neonatal population, at present only neonates with GA of at least 36 weeks receive TH. Nevertheless, also preterm infants can present with HIE, but the symptoms and pathology are different, including subcortical gray matter injury combined with white matter damage. Studies in preterm fetal sheep indicate that moderate cerebral hypothermia can reduce both white and gray matter injury. Clinical reports on preterms receiving hypothermia provide concerns about adverse effects, morbidity, and mortality (Rao et al., 2017; Herrera et al., 2018). Due to these concerns, it is suggested to first investigate TH in systematic trials in late preterms (GA 31–36 weeks) with a clinical picture in line with term cases suffering from HIE (Gunn and Bennet, 2008). A randomized controlled trial including preterms (GA 33–35 weeks) with moderate to severe encephalopathy, investigating TH, is ongoing (Clinicaltrials.gov NCT01793129). For preterm neonates with necrotizing enterocolitis with multiple organ dysfunction syndrome, Hall et al. reported that mild hypothermia for 48 h was feasible and safe. Additional study on the efficacy of hypothermia for this special subpopulation is certainly needed (Hall et al., 2010). When hypothermia PBPK models would be applied for dose selection in these subpopulations or new indications, the models should be completed with system ontogeny data and covariates of the respective population and/or disease condition. In general, controlled temperature in clinical care becomes more popular, but the impact on drug disposition often remains underexplored. Within adult medicine, hyperthermic intraperitoneal chemotherapy (HIPEC, optimal temperature range 41–43°C) is used in oncology treatment (Kusamura et al., 2008). Surgery (to eliminate all macroscopic disease) and HIPEC (to target residual microscopic disease) combined have been recognized as therapy of a subgroup of patients with *e.g.* peritoneal carcinomatosis. However, different regimens of intraperitoneal chemotherapy exist, and standardization in methodology for administration of this therapy is lacking (Valle et al., 2016). PBPK models might also be helpful to gain insight in drug disposition in these indications.

## Future Directions and Conclusion

We recommend sharing the data (PK, systems ontogeny) and models in open source platforms in order to make progress in the field of thermo-pharmacology and to support the development of temperature-driven PBPK models. This allows research collaborations, not only for PBPK model development, but also for validation efforts. Finally, besides PK, also research on PD during hypothermia is needed. When drug exposure can be predicted, the concentration–effect relationship needs further attention. Also, for PD targets, (*e.g.* receptor expression and activity), maturational as well as nonmaturational covariates need to be taken into account, explaining variability in efficacy of pharmacotherapy between and within patients. Study of PD in neonates is challenging due to the lack of reference values (*e.g.* biomarkers, vital parameters like blood pressure) and consensus on definitions. Development of adequate nonclinical models to investigate neonatal PD during PA and TH is of relevance.

In conclusion, this review summarized current evidence on the impact of PA and TH on neonatal drug disposition and also identified several knowledge gaps in this field. In order to enable precision dosing during PA and TH, we proposed the development of a robust PBPK framework by using a multidisciplinary, translational approach. The applicability of the proposed workflow and the challenges in the development of such a PBPK framework were illustrated for midazolam as a model drug. However, this PBPK framework is clinically relevant in the broad field of thermo-pharmacology, and recommendations for future directions were provided.

## Author Contributions

All authors (AS, PA, SV, and KA) contributed to the writing and reviewing of the article.

## Funding

The research is supported by a FWO grant (G0D052N, Fonds voor Wetenschappelijk Onderzoek) of the Flemish government. The research activities of AS are further supported by the Clinical Research and Education Council of the University Hospitals Leuven.

## Conflict of Interest

The authors declare that the research was conducted in the absence of any commercial or financial relationships that could be considered as a potential conflict of interest.
